# Clinical Outcomes of Injectable Porcine Collagen in Diabetic Patients with Achilles Tendinopathy: A Retrospective Study

**DOI:** 10.3390/jcm15041381

**Published:** 2026-02-10

**Authors:** Giacomo Placella, Niccolò Biavardi, Mattia Alessio Mazzola, Vincenzo Salini

**Affiliations:** San Raffaele University, 20132 Milan, Italy; biavardi.nicolo’giuseppe@hsr.it (N.B.); malessiomazzola@gmail.com (M.A.M.); salini.vincenzo@hsr.it (V.S.)

**Keywords:** diabetic patients, Achilles tendinopathy, pain, VAS, VISA-A, porcine collagen injection, BMI, return to work

## Abstract

**Background/Objective**: Achilles tendinopathy (AT) is a disabling condition, and treatment options are limited in patients in whom corticosteroid injections are discouraged or contraindicated, including individuals with diabetes. Porcine collagen injections have been proposed as a conservative option; however, clinical evidence in diabetic populations remains limited. This study aims to describe pain and functional outcomes after peritendinous collagen injections in diabetic patients with chronic Achilles tendinopathy. **Methods**: Twenty-two diabetic patients with ultrasound-confirmed degenerative Achilles tendinopathy refractory to conservative management were retrospectively included and split into two groups according to AT type: insertional (IAT) and non-insertional/midportion (NIAT). All patients received five weekly ultrasound-guided peritendinous collagen injections. Outcomes included VAS assessed at baseline, after the second injection, at 1 month, and at 6 months; VISA-A at baseline and 6 months; return-to-work (RTW) time; and adverse events. Baseline variables included BMI, HbA1c, symptom duration, and previous treatments. Analyses were based on descriptive statistics and within-group comparisons over time. **Results**: All patients completed the treatment protocol, and no adverse events were recorded. Pain significantly improved over follow-up in both subgroups. Mean VAS decreased from baseline to 6 months (mean ΔVAS: 5.1 in IAT and 4.4 in NIAT; *p* = 0.001 for within-group change). VISA-A also improved at 6 months (mean ΔVISA-A: 32.78 in IAT and 38.97 in NIAT; *p* < 0.0001). Median RTW was 37 days in IAT and 35 days in NIAT (*p* > 0.05). No significant between-group differences were observed for VAS or VISA-A changes (*p* > 0.05). **Conclusions**: In this uncontrolled retrospective case series, peritendinous collagen injections were feasible and well-tolerated in diabetic patients with Achilles tendinopathy and were associated with clinically relevant improvements in pain and functional outcomes at 6 months. These findings are hypothesis-generating and warrant confirmation in prospective controlled studies.

## 1. Introduction

The Achilles tendon in medicine is also known as the “triceps surae”. It is the strongest and thickest tendon in the entire human body [1]. This tendon connects with the aponeuroses of the gastrocnemius, soleus, and plantaris muscles and joints them to the calcaneus bone [2]. The outer sheath of the tendon is a sheath-like structure made up of a single layer of cells; it is not a true synovial sheath, but rather a “false sheath” called the paratenon [2,3,4]. The paratenon significantly contributes to providing blood supply to the tendon [4]. The Achilles tendon is essential for allowing the calf muscles to exert force on the heel; this function becomes crucial for walking and running correctly [5,6,7]. The Achilles tendon can be affected by various pathological conditions such as insertional [2,8] and non-insertional tendinitis [9,10], paratenonitis [11,12] and tendon rupture [13,14]. Tendinopathy is one of the most common conditions affecting the Achilles tendon. The factors and causes triggering Achilles tendinopathy (AT) are divided into two main groups: intrinsic factors and extrinsic factors [5,6,7]:

- Intrinsic factors:
  These include anatomic factors, age, sex, metabolic dysfunction [15] foot cavity, dysmetria, muscle weakness, imbalance, gastrocnemius dysfunction [16], anatomical variation in the plantaris muscle [17], tendon vascularization [18] and torsion of the Achilles tendons [19].
- Extrinsic factors:
  These include mechanical overload, constant effort [20], diabetes and obesity [21,22,23,24,25,26] medications (corticosteroids, anabolic steroids, fluoroquinolones) [27,28,29,30], hard training surfaces and direct trauma to the lower leg [31].

The pathophysiology of AT, described as the anatomical abnormality of the tendon, is characterized by a reduction in parallel type I collagen fibers, associated with infiltration of adipose tissue and proliferation of vascular tissue. These changes manifest as a thickening of the tendon, visible through conventional imaging diagnostics [2,32]. Furthermore, the Achilles tendon histopathology showed its composition of 95% type I collagen fibers; these fibers provide strength and flexibility to the Achilles tendon. A decrease in this type of fiber can also occur with the normal aging process or as result of injuries. In tendinopathy, which includes both tendinosis and tendinitis, the amounts of proteoglycans, water content, and disorganized type III collagen, unlike type I, increase [32]. Notably, it has been shown that an increase in advanced glycation end-products (AGEs) in association with diabetes pathology impacted the functionality of the Achilles tendon [33]. The changes concerning the properties and the content of collagen fibers have been associated with diabetes [23,24,25]. These morphological changes observed in tendons can also be present in the early stages of type 2 diabetes (T2DM). Since the diagnosis of T2DM is often made when some patients already show evidence of chronic complications, this implies that the effects of hyperglycemia would have already been exerted for some time on tendon structures [34,35]. Alterations in the fibrillar organization of the Achilles tendon, the presence of hypoechoic areas found on ultrasound imaging, and calcific formations within the tendon are common degenerative abnormalities [35,36].

ATs can be categorized into two groups [5]: Insertional (AT-I) [22,24] and non-insertional (AT-NI) [6]. Achilles tendinopathies are pathological metabolic conditions frequently found in T2DM patients [22,33,34]. These pathologies are characterized by the onset of chronic pain, which leads to progressive motor disability both in normal daily activities and during sports activities. Therefore, therapeutic effectiveness in relieving pain and improving functionality is the desired goal for both patients and specialists in this field. The scenario concerning the management of AT is very broad and complex. Physic-kinesis-therapy (FKT) always constitutes an initial starting point for AT treatment [35]. However, clinicians, responsible for selecting the best clinical treatment for the patient, often must choose between an interventional approach (surgery) and a non-interventional approach (conservative therapy). The choice of a non-surgical approach is always preferred over a surgical one, which is generally considered only in cases of Achilles tendon rupture or in instances where conservative treatment has failed. Fortunately, beneficial effects have been reported for the treatment of AT and tendinopathies in general through various conservative approaches, including the use of platelet-rich plasm [36,37], shockwaves [38,39,40], and porcine collagen. This type of treatment has been successfully tested in various pathological conditions of the musculoskeletal system [41], such as greater trochanteric pain syndrome (GTPS) [42,43], knee osteoarthritis (K-OA) [44,45,46], hip tendinitis [47,48,49] and tendinopathies of the rotator cuff of the shoulder [47]. Nonetheless, it has been shown that collagen injection collaborates with tenocytes, demonstrating its relationship with mechanical stimuli [42,43]. Although diabetes is associated with glycation-related collagen cross-linking and structural disorganization within tendon tissue, collagen injections should not be interpreted as a direct reversal of established AGE-mediated changes. The rationale for peritendinous collagen supplementation is instead based on biological plausibility: diabetes impairs tendon homeostasis and collagen turnover, with altered extracellular matrix remodeling and tenocyte function. Providing exogenous collagen in the peritendinous environment may act as a supportive extracellular matrix substrate and modulate the local microenvironment, potentially facilitating reparative processes and symptom improvement. However, direct mechanistic evidence supporting these pathways in diabetic Achilles tendinopathy remains limited and warrants dedicated experimental investigation.

Having said all that, the aim of this study is to evaluate the effectiveness of collagen infiltration in Achilles tendons in diabetic subjects for the improvement in pain symptoms and functionality.

## 2. Materials and Methods

### 2.1. Study Design, Data Collection, and Eligibility Criteria

This study is a single-center, uncontrolled retrospective case series including diabetic patients treated for Achilles tendinopathy during 2024. Clinical and demographic data were retrospectively collected by two authors (G.P. and M.B.) and entered into a dedicated anonymized database. For confidentiality, each patient was assigned an encrypted alphanumeric code and all identifiable information was removed before analysis (during the first part of 2025).

Inclusion Criteria

Patients were eligible if they met all of the following criteria:

▪ Age ≥ 18 years;
▪ Pre-existing diagnosis of diabetes mellitus documented in medical records, with ongoing treatment when applicable;
▪ Baseline metabolic status documented by HbA1c level when available (collected as a descriptive variable, not as a diagnostic criterion);
▪ Symptomatic degenerative Achilles tendinopathy confirmed by clinical assessment and ultrasound examination;
▪ Persistence of pain and functional impairment for ≥6 months despite conservative management;
▪ Regular physical activity and/or recreational sport participation documented in clinical history (when available);
▪ Continuous pain and functional limitation affecting daily activities and/or sports participation;
▪ No previous surgical treatment for Achilles tendinopathy.

Diabetes Type

Diabetes type (type 1 vs. type 2) was retrieved from clinical records when available. The cohort consisted predominantly of patients with type 2 diabetes.

Exclusion Criteria

Patients were excluded in cases of:

▪ Partial or complete Achilles tendon rupture;
▪ Previous surgical treatment of the Achilles tendon;
▪ Previous infiltrative or regenerative treatments for Achilles tendinopathy;
▪ Prior corticosteroid injections for Achilles tendinopathy;
▪ Known hypersensitivity or declared allergy to collagen or related derivatives.

### 2.2. Ultrasound Assessment and Definition of Tendinopathy Subtypes

All patients underwent musculoskeletal ultrasound as part of routine clinical assessment to confirm the diagnosis of degenerative Achilles tendinopathy and to guide the injection procedure. Ultrasound was used to localize the affected tendon segment and the area of maximal structural alteration, typically characterized by tendon thickening and focal loss of the normal fibrillar echotexture, with or without peritendinous changes.

For analytical purposes, Achilles tendinopathy was classified by anatomical location based on clinical and ultrasound findings:

▪ Insertional Achilles tendinopathy (IAT): pain and sonographic alterations located within 0–2 cm from the calcaneal insertion;
▪ Non-insertional (midportion) Achilles tendinopathy (NIAT): involvement of the tendon portion located 2–7 cm proximal to the insertion.

### 2.3. Treatment Protocol and Injection Technique

All patients received a standardized treatment protocol consisting of five weekly peritendinous porcine collagen injections (MD-Tissue, Medical Device, Guna spa, Milan, Italy). Each injection session consisted of 2 mL of collagen, for a total cumulative dose of 10 mL (5 vials).

All procedures were performed under ultrasound guidance using a 22G needle (23 mm length), to ensure accurate peritendinous placement and to avoid intratendinous injection. The target area was disinfected using alcohol-based antiseptic solution (or equivalent), and the procedure was conducted under sterile conditions. The collagen was injected around the tendon at the level of the symptomatic region (insertional site and/or proximal course), distributing the injectate within the peritendinous/periparatenon space under real-time ultrasound monitoring. Local anesthetic was not routinely administered.

### 2.4. Post-Injection Management and Concomitant Treatments

Given the retrospective design, post-injection management and concomitant interventions were extracted from clinical charts. Patients were generally advised to follow activity modification after injection and to avoid additional infiltrative procedures during the treatment period.

To address potential confounding, the presence of co-interventions during follow-up was documented when available, including physiotherapy, structured loading/exercise programs, footwear modification/orthoses, use of NSAIDs or analgesics, extracorporeal shockwave therapy, and any relevant changes in diabetes management. These variables were collected to improve interpretability of outcomes in the absence of a control group.

### 2.5. Endpoints and Safety Monitoring

The primary objective of this study was to evaluate the clinical evolution of pain and function at 6 months after treatment completion. The secondary objective was to monitor safety and tolerability of collagen injections.

Adverse events potentially attributable to collagen were recorded from clinical documentation. Events of interest included persistent post-procedural pain, skin rash, swelling/edema, and allergic reactions.

### 2.6. Clinical Outcome Measures

Pain intensity was assessed using the Visual Analog Scale (VAS), ranging from 0 (“no pain”) to 10 (“worst pain imaginable”). Functional status was assessed using the Victorian Institute of Sport Assessment–Achilles questionnaire (VISA-A), a validated patient-reported outcome measure for Achilles tendinopathy assessing pain, function in daily life, and sport activity. VISA-A scores range from 0 to 100, with 100 indicating absence of symptoms and full function.

VAS and VISA-A were collected at baseline (T0, before the start of treatment) and at follow-up (T1, 6 months after the end of the treatment cycle). Return-to-work (RTW) and return-to-sport (RTS) timings were recorded when available. The RTW was defined as the time elapsed from the start of treatment (T0; first injection) to the first documented date of resumption of the patient’s usual work activity. Time was recorded in days/weeks based on clinical documentation and follow-up reports.

### 2.7. Statistical Analysis

Continuous variables were summarized using mean ± standard deviation (SD) or median (minimum–maximum), as appropriate. Categorical variables were reported as counts and percentages based on the non-missing sample size.

Normality was assessed using the Shapiro–Wilk test. Changes between baseline (T0) and follow-up (T1) were assessed using paired Student’s t-test for normally distributed data. Subgroup analyses between insertional and non-insertional tendinopathy were performed using ANOVA on delta (change) VAS and VISA-A outcomes.

Time-to-event analysis for RTW was evaluated using Kaplan–Meier survival curves with log-rank testing and hazard ratio (HR) estimates with 95% confidence intervals (CI), when applicable. Statistical significance was set at *p* < 0.05.

Statistical analyses were performed using GraphPad Prism (version 10.0, San Diego, CA, USA). Data were recorded in a dedicated spreadsheet (e.g., Microsoft Excel), and original rows of anonymized data can be made available upon reasonable request.

## 3. Results

### 3.1. Demographic and Clinical Data

**Figure 1:**
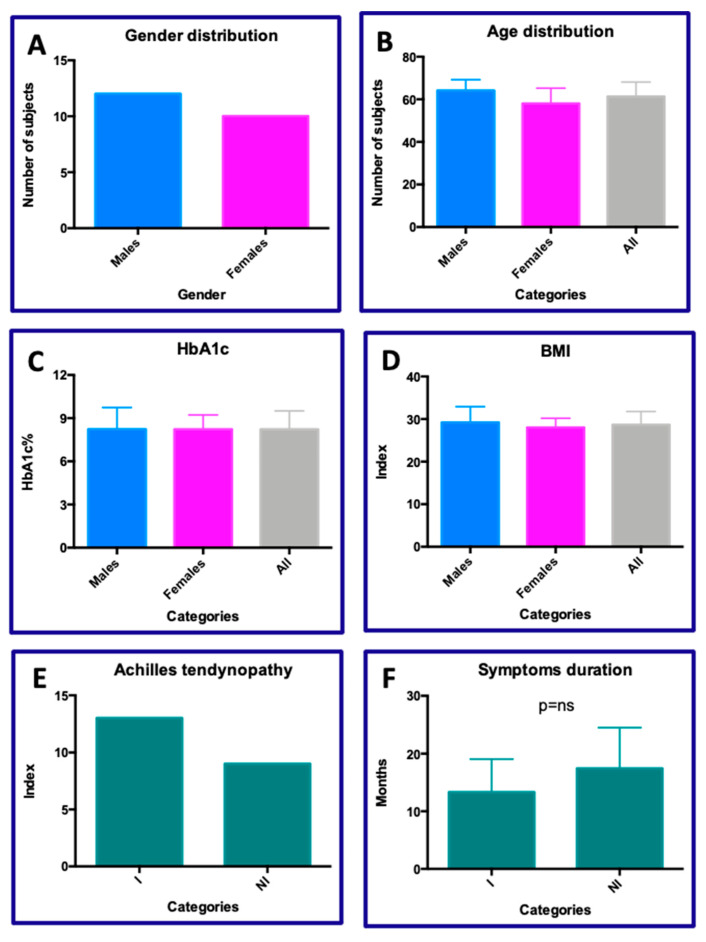
Demographic and clinical data. No statistical different distributions were observed according to the following sets: (**A**) gender; (**B**) age; (**C**) HbA1c; (**D**) BMI; (**E**) type of tendinopathy—insertional (I) and non-insertional (NI); (**F**) symptom duration (months).

The demographic and clinical data of the 22 patients included in the study are reported in Figure 1. No statistically significant differences were observed regarding the distribution of gender (12 M vs. 10 F; Figure 1A) and age (64.1 ± 5.1 M vs. 58.0 ± 7.2 F vs. 61.3 ± 6.7 All; Figure 1B) among the enrolled subjects. All subjects were classified as diabetic patients based on the distribution of glycated hemoglobin values (8.2 ± 1.5 M vs. 8.2 ± 0.9 F vs. 8.2 ± 1.2 All; Figure 1C; *p* = 0.9911) and body mass index (BMI) values (29.1 ± 3.7 M vs. 28.1 ± 2.1 F vs. 28.6 ± 3.1 All; Figure 1D; *p* = 0.8004). From a clinical perspective, patients were divided based on the type of Achilles tendinopathy (ATs; Figure 1E) into two groups: insertional (I; *n* = 13) and non-insertional (NI; *n* = 9). The duration of symptoms was recorded for each patient and no statistically significant differences were found between subjects with AT-I and those with AT-NI (13.3 vs. 17.4; Figure 1F; *p* = 0.1477). Collagen treatment was applied equally to both groups, which were shown to have homogeneous characteristics.

### 3.2. VAS Results

**Figure 2:**
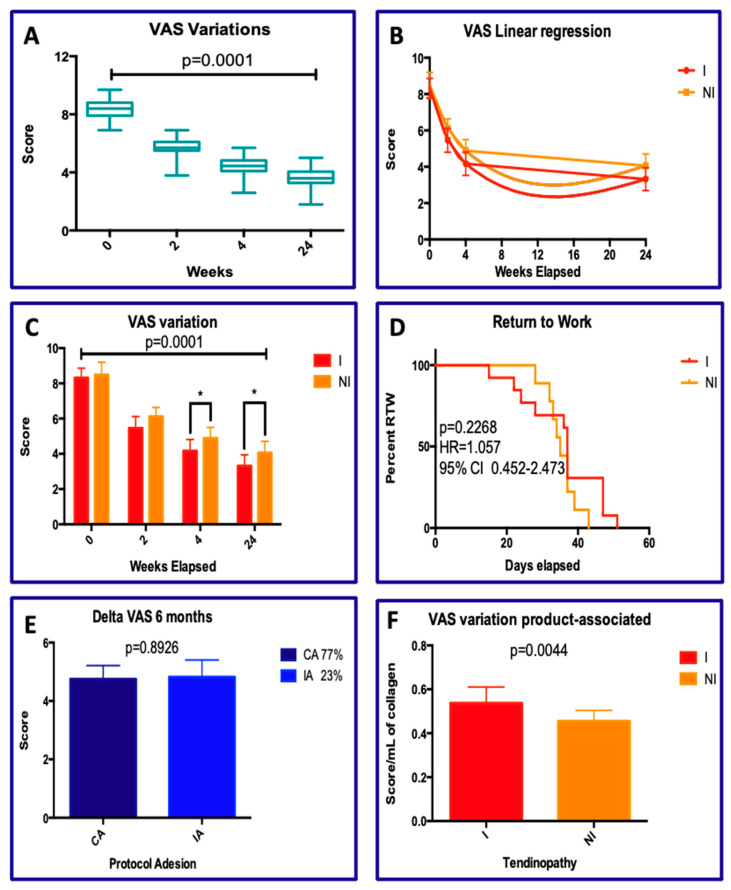
VAS analyses. (**A**) Mean VAS values including all patients during the time; (**B**) VAS linear regression according to the type of AT—insertional (I) and non-insertional (NI); (**C**) ANOVA test concerning the VAS variation in AT-I and AT-NI groups; (**D**) log-rank test for the RTW time calculation; (**E**) delta VAS according to the compliance of protocol; (**F**) delta VAS variation associated for mL of collagen injected. The asterisk symbol “*”, indicates statistical significance (*p* < 0.05) between the signed groups.

The positive changes in VAS (Figure 2) were observed as early as two weeks and increases up to the twenty-fourth week (Figure 2A; *p* < 0.0001). The pattern of VAS change follows a consistent hyperbolic trend, both for patients with TA-I and for those with TA-NI. The curves are parallel to each other, with a slight predominance in the TA-I patient group (Figure 2B). These differences remain consistent between the two curves throughout the treatment period and the differences at each step (2, 4, and 24 weeks) are statistically significant for both groups over time (Figure 2C; *p* < 0.0001).

This is in agreement with the treatment revealing similar clinical results between the two groups regarding the return-to-work interval (RTW; Figure 1D), where no statistically significant differences were observed between TA-I and TA-NI groups. The average differences (measured in days for RTW) were, respectively, 35 vs. 37 days (*p* = 0.2268; HR = 1.057). The protocol provided the administration of five doses of porcine collagen, each 2 ml (A total of 10 mL, MD-Tissue). The compliance with the protocol was complete in 77% of cases (complete adherence CA; 5 doses), while incomplete adherence (IA; 4 doses) was observed in 23% of cases. However, the change in VAS at six months was not statistically significant between the two groups (Figure 2E; *p* = ns). Even with four doses, the treatment’s efficacy was demonstrated. Finally, the improvement in VAS in terms of score points per ml of collagen applied was a little better in patients with TA-I vs. TA-NI (Figure 2F; *p* = 0.0044), although the average infiltrated volumes were identical (9.38 vs. 9.78).

### 3.3. VISA-A Results

**Figure 3:**
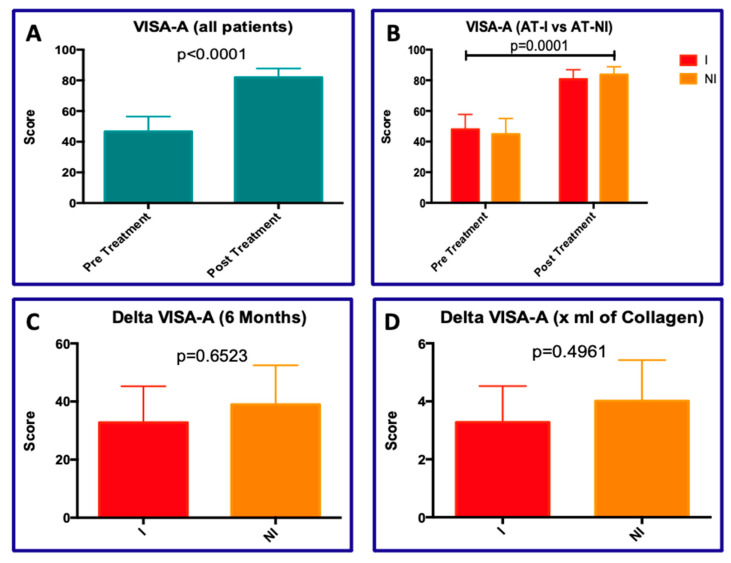
VISA-A analyses. (**A**) VISA_A mean scores in all AT patients pre- and post-treatment; (**B**) VISA-A variation in the AT-I and AT-NI; (**C**) delta VISA-A variation after six months; (**D**) delta VISA-A variation for ml of collagen administered.

The statistical analysis concerning the VISA-A score (Figure 3) revealed a significant variation in mean values comparing pre- vs. post-treatment in all patients (45.50 vs. 82.70, *p* < 0.00001; Figure 3A). Indeed, ANOVA analysis looking for the VISA-A variation in TA-I vs. AT-NI pre- and post-treatment showed significant difference for the treatment (*p* < 0.0001; Figure 3B), while no statistically significant differences were observed between the two types of tendinopathies (*p* = 0.2222; Figure 3B). The delta VISA-A scores (at a 6-month follow-up) were equal to 32.78 vs. 38.97 for AT-I and AT-NI, respectively (*p* = 0.6523; Figure 3C). Numerical change of VISA-A per unit of collagen injected in the two types of ATs (I vs. NI) were 3.28 vs. 4.01, respectively (*p* = 0.4961; Figure 3D). Although in this last analysis the differences are not statistically significant, AT-NI patients seem to benefit more from collagen treatment.

### 3.4. Adverse Events (AEs)

Throughout the duration of this study, no adverse events associated with collagen administration were reported, among those included and reported in the Section 2.

## 4. Discussion

Achilles tendinopathy is associated with external mechanical factors such as prolonged or repetitive loading; however, systemic comorbidities may also contribute to tendon vulnerability by altering collagen composition and microstructure [7,50]. Intrinsic factors—including body mass index (BMI), diet, metabolic disorders, and foot morphology (e.g., cavus foot)—have been reported as risk factors for tendinopathies, including Achilles tendinopathy [6,51]. Diabetes, in particular, has been linked to tendinopathy through metabolic and structural mechanisms, including altered cellular metabolism, increased intracellular water content and edema, reduced tolerance to ischemic stress, and accumulation of advanced glycation end-products that increase collagen cross-linking and modify tissue architecture [6,33,51]. Tendon microstructural disorganization may also be influenced by dyslipidemia or hypercholesterolemia, potentially leading to lipid deposition and xanthoma formation [7]. In diabetic patients, Achilles tendinopathy may therefore represent a chronic condition with variable clinical course and long-term morbidity.

Initial management of Achilles tendinopathy is generally conservative, and many patients show satisfactory outcomes with non-operative approaches [6,7]. Physiotherapy and structured loading/kinesitherapy are typically considered first-line conservative strategies, while surgery is reserved for refractory cases [8,52,53]. Several local conservative treatments have also been investigated, including platelet-rich plasma [36,37], extracorporeal shockwave therapy [38,39,40], and collagen-based injections [41,42,48,49,54]. Although collagen formulations have been explored in other tendinopathies, their use in both insertional and non-insertional Achilles tendinopathy remains relatively limited.

In the present uncontrolled retrospective case series, collagen injections were associated with an improvement in pain and function over follow-up in diabetic patients affected by both insertional and non-insertional Achilles tendinopathy, as documented by VAS (Figure 2) and VISA-A (Figure 3). From a mechanistic standpoint, collagen supplementation may be biologically plausible in diabetic tendinopathy, given the altered collagen metabolism and structural changes induced by glycation [6,7,33,55]. Moreover, in vitro evidence suggests that collagen may modulate tenocyte regenerative and morpho-functional activities [56,57]. Nevertheless, due to the absence of a control group, the observed clinical improvements cannot be attributed exclusively to collagen treatment, and may partially reflect natural history, regression to the mean, or other non-specific effects.

The magnitude and temporal pattern of improvement observed in this cohort appear broadly consistent with prior reports of conservative approaches such as platelet-rich plasma [36] and extracorporeal shockwave therapy [40]. Collagen injections may therefore represent a feasible conservative option in selected diabetic patients, particularly in clinical contexts where corticosteroid injections are discouraged or contraindicated [6,28,29,58], despite isolated reports of steroid efficacy [59]. Other pharmacological approaches (e.g., antibiotics) are not recommended for tendinopathy management, even in non-diabetic patients [10,27,30,37,60,61].

Key observations from the present case series

Based on longitudinal outcomes, we observed the following clinically relevant patterns:

▪ A reduction in pain (VAS) was already detectable at 2 weeks and continued to improve up to 24 weeks.
▪ Improvements were observed in both insertional and non-insertional Achilles tendinopathy subgroups.
▪ A small subset of patients received four injections instead of five. Although improvement was also noted in this subgroup, the sample size was limited and the study was not designed or powered to evaluate dose–response relationships. Therefore, no conclusion can be drawn regarding a minimum clinically effective dose, and this finding should be considered exploratory.
▪ Patients with insertional Achilles tendinopathy showed a trend toward greater pain improvement; however, functional outcomes (VISA-A) did not differ significantly between subgroups.

Overall, this retrospective case series supports the feasibility and safety of infiltrative collagen treatment in diabetic patients with Achilles tendinopathy, with a signal of improvement in pain and function over follow-up. However, several limitations must be considered: (1) the small sample size reduces statistical power and limits subgroup comparisons; (2) outcomes were assessed up to six months, and longer follow-up would help determine durability of response; and (3) imaging-based evaluation of tendon remodeling was not systematically used to correlate clinical improvement with structural changes. Despite these limitations, high adherence to the protocol and consistent outcome tracking strengthen the descriptive value of these findings and support future prospective controlled investigations.

## 5. Conclusions

Porcine collagen injections were feasible and well-tolerated in patients with diabetes affected by Achilles tendinopathy. In this uncontrolled retrospective case series, patients showed a clinically relevant improvement over follow-up, including reduced pain and enhanced functional outcomes, both in insertional and non-insertional presentations. Although diabetes-related alterations in tendon collagen metabolism provide a biological rationale for supplementation with exogenous collagen, the present study design does not allow causal inference or assessment of comparative effectiveness, and the observed improvements may also reflect natural history, regression to the mean, and non-specific effects. Therefore, collagen injections (MD-Tissue) may represent a promising conservative option, particularly in settings where corticosteroids are discouraged or contraindicated. Prospective controlled multicenter studies are warranted to confirm these findings, define the optimal dosing schedule, and investigate factors associated with treatment response.

## Data Availability

The raw data from the study will be made available upon request from the corresponding author of this study: Giacomo Placella.
